# Patient-Centered Communication Behaviors That Correlate With Higher Patient Satisfaction Scores

**DOI:** 10.1177/2374373517750414

**Published:** 2018-01-15

**Authors:** Doug Finefrock, Sridhar Patel, David Zodda, Themba Nyirenda, Richard Nierenberg, Joseph Feldman, Chinwe Ogedegbe

**Affiliations:** 1 Hackensack University Medical Center, Hackensack, NJ, USA; 2 Kaiser Permanente, Oakland, CA, USA; 3 St. George’s University School of Medicine, St. George’s, Grenada, West Indies

**Keywords:** patient satisfaction, patient/relationship centered skills, clinician–patient relationship, communication, emergency medicine, empathy, patient feedback, service excellence

## Abstract

**Background:**

With increased emphasis on improving the patient experience, clinicians are being asked to improve their patient-centered communication behaviors to improve patient satisfaction (PS) scores.

**Local Problem:**

The relationship between clinician communication behaviors and PS is poorly studied in the emergency department (ED) setting. The purpose of this study was to identify whether specific communication behaviors correlate with higher PS scores in the ED setting.

**Methods:**

During a quality improvement project, we performed 191 bedside observations of ED clinicians during their initial interaction with patients and recorded the frequency of 8 positive communication behaviors as defined by the PatientSET tool.

**Interventions:**

The frequency of use of the PatientSET communication behaviors was compared between known high performers in Press Ganey PS scores versus low performers.

**Results:**

Being a high Press Ganey performer was associated with a significantly higher frequency of performance in 6 of the 8 PatientSET communication behaviors.

**Conclusions:**

Positive communication behaviors such as those in the PatientSET tool occurred more frequently in ED clinicians with higher PS scores.

## Introduction

As hospitals strive to improve their overall patient satisfaction (PS) scores, there is increased administrative attention on the emergency department (ED) experience where patients often form their first impression of the overall hospital experience. This has led to scrutiny of ED PS scores and accountability for ED clinicians to improve their individual PS scores, often without the necessary support or guidance. Hospital efforts to improve ED PS tend to focus on operational improvements or the physical nature of the ED rather than individual clinician communication training (1). In addition, there is a dearth of evidence demonstrating what specific communication behaviors are associated with higher PS scores.

Clinician communication training efforts have focused on the undergraduate and graduate medical education setting rather than training for practicing physicians (2). In a standardized patient setting, resident physician’s positive nonverbal skills were associated with greater initial PS (2). Although there has been some improvement in PS after communication training, “most training programs after medical school do not include communication skills in their curricula” (3). As PS scores become increasingly important, communication training may consistently begin in medical school and extend throughout clinical practice.

Unfortunately, it is not known what behaviors should be included in communication programs to improve PS scores. Several different independent physician behaviors are thought to improve PS. Behaviors such as properly identifying yourself and maintaining eye contact have been shown to improve patient perceptions of trust (4).

Although many ED physicians are being evaluated by PS scores, the majority of emergency medicine (EM) residencies do not provide PS data or have a PS curriculum to help prepare future EM physicians (5). There have been attempts to create PS surveys specifically designed for EM residents to prepare them for life as an attending physician (6). Currently, the American College of Emergency Physicians has issued an information paper that describes PS surveys and suggests positive behaviors such as sitting down during the interaction, using an appropriate greeting, managing expectations, and providing diversions (7). The data supporting these behaviors and the relative importance of each behavior, however, have not been described.

The objective of this patient-centered quality improvement (QI) study was to determine whether specific clinician patient-centered communication behaviors correlate with higher PS scores in the ED setting by comparing the frequency of these behaviors between ED clinicians with high versus low PS scores.

## Methods

This study is a retrospective review of a QI initiative in the adult section of a large, tertiary care, community teaching ED, in northeast New Jersey with an annual census of 120,000 patients staffed by 37 ED clinicians. It occurred over a 10-month period from December 2012 through October 2013. No EM residents saw patients during this period. Non-EM rotating residents saw an estimated 1% of the patients during this period. The study included EM clinicians with N>30 Press Ganey (PG) surveys from the previous year and excluded those with N<30 PG surveys. High performers were arbitrarily defined as having PG scores >40th percentile, while low performers were defined as having PG scores <40th percentile.

### Relevant QI Tools

#### Press Ganey survey scores

The PG PS instrument is a proprietary survey and data instrument that asks patients to rate their ED experience. The PG ratings include, for example, whether the physician took time to listen as well as their courtesy and concern for patient comfort. These ratings are collected, then compared to and ranked against ratings from other similar departments with a database that now includes a large number of medical centers. Clinicians can be identified and their individual rankings assigned a percentile rank compared to clinicians in similar hospitals. Press Ganey recommends that clinicians should have at least 30 PS surveys before assessing performance in PS. On the basis of these percentile rankings, clinicians can be categorized as high or low performers. Press Ganey was selected by the hospital as the sole company that obtains our PS surveys. It was the only means for us to determine our department’s PS scores for this study. We presently use the same survey tool and physician questions that were used at the time of the study.

#### PatientSET “Satisfaction Every Time” communication training program

This tool was chosen because it was created by one of our authors to improve clinician bedside manner. None of the other authors or study participants were involved in the formation of this program. The program includes the following 8 observable communication behaviors:

1. Pause before entering.
2. Smile and make eye contact.
3. Introduce.
4. Shake hands.
5. Acknowledge the wait and apologize for it.
6. Begin with open-ended question like “How can I help you?”
7. Do at least 1 nonmedical gesture.
8. Overestimate time.

### Measures

Of the 37 total ED clinicians, 19 (16 attending physicians, 2 physician assistants, and 1 advanced nurse practitioner) met the inclusion criteria for having N > 30 PG surveys from the 4 quarters in the preceding year. The clinicians included had an average of 48 PG surveys each. All of the patients in these surveys were treated by the ED clinician in the ED and discharged from the ED without being admitted to the hospital. Of the 19 ED clinicians, 8 were defined as high PG performers and 11 as low PG performers using the 40th percentile ranking arbitrary cutoff based on the site average scores. These clinicians had an average PG score in the 37th percentile. The high PG performers had an average PG score in the 69th percentile (range: 44th to 98th percentile), while the low PG had an average PG score in the 14th percentile (range: 1st to 37th percentile). The high PG performers had an average of 13.9 years since they initially received EM board certification compared to 12.1 years for the low PG performers. The high PG performers included 5 males and 3 females, while the low PG performers included 6 males and 5 females. All clinicians were predominately day and evening shift workers, except 2 in the low PG group who worked predominantly night shifts. The observations occurred during daytime hours, except for the 2 night shift workers. All clinicians were told they would be observed for patient interactions and bedside manner. They were blinded to the study hypothesis.

We performed 191 total bedside observations on these 19 ED clinicians chosen by convenience. Observed behavior data were collected via a standard electronic data collection tool by members of the ED service excellence team. This included nurses, patient care associates, and social workers, all familiar to the ED. Each observer completed the online PatientSET training prior to their observations then were randomly assigned ED clinicians to observe. They were blinded to the study hypothesis and prior clinician PG scores. A positive communication behavior was recorded as “yes” or “no” to represent present or absent. After the observations, all clinicians were given the PatientSET online training as a departmental QI initiative to improve PS.

Comparison of the number of times an ED clinician exhibited a particular positive behavior was considered as a Poisson process, that is, discrete count of the occurrence of each positive behavior for each clinician. Associations of the number of times the clinicians exhibited the positive communication behavior were examined using Poisson regression analysis. Summaries were presented as count (percentage).

An analysis was conducted to compare the number of times clinicians exhibited positive behaviors between high PG performers and low PG performers. Results of the Poisson regression analysis were reported as rate ratio (RR), 95% confidence interval (CI) of RR, and *P* value. An RR > 1 and 95% CI that excluded 1 indicated that the grouping variable was associated with increased likelihood of exhibition of positive behavior. An RR < 1 and 95% CI that excluded 1 indicated that the grouping variable was associated with decreased likelihood of exhibition of positive behavior. Poisson regression analysis was performed by utilizing PROC GENMOD SAS 9.4 with distribution=Poisson link=log. Any *P* < .05 was considered statistically significant. All data analysis was conducted using SAS 9.4 (SAS Institute Inc, Cary, North Carolina).

## Results

Our results compared the frequency of PatientSET communication behaviors between 8 high PG performers and 11 low PG performers. The high performers had an average PG score of 69% (N = 412 total PG surveys), while the low performers had an average PG score of 14% (N = 491 total PG surveys). Each clinician had an average of 10.1 observations per clinician, with a range of 9 to 11 observations per clinician.

### Comparative Analysis of PatientSET Behaviors in Low Versus High PG Performers

We compared PG performance from the prior 4 quarters and the number of times the clinicians exhibited specific behavior using Poisson regression analysis. The results are presented in Table 1. Except for two behaviors “pause before entering” and “introduce self and others,” the rest of the 6 behaviors was exhibited more frequently by the high PG performers compared to the low PG performers.

**Table 1.** Association Between Low and High PG Performing Clinicians and Specific Behaviors.^a^

Item	High PG (n = 8)/Low PG (n = 11)	RR (95% CI)	*P* Value
Pause before entering	93%/78%	1.20 (0.88-1.63)	.2504
**Smile and make eye contact**	**99%/65%**	**1.55 (1.13-2.13)**	**.0073**
Introduce self and others	93%/98%	0.95 (0.71-1.28)	.7586
**Shake hands**	**74%/47%**	**1.56 (1.07**-**2.26)**	**.0194**
**Acknowledge the wait and apologize**	**44%/3%**	**16.76 (5.13**-**54.74)**	**<.0001**
**Begin with “How can I help you?”**	**88%/35%**	**2.50 (1.69**-**3.70)**	**<.0001**
**Do at least 1 nonmedical gesture**	**85%/21%**	**4.13 (2.57**-**6.61)**	**<.0001**
**Overestimate time**	**75%/11%**	**6.88 (3.70**-**12.78)**	**<.0001**

Abbreviations: CI, confidence interval; PG, Press Ganey; RR, rate ratio.

^a^Results in bold text indicate items that were statistically associated with the PatientSET communication program intervention.

A high PG performer was 16.76 times more likely to acknowledge the wait and apologize than a low performing PG clinician (RR = 16.76, 95% CI: 5.13-54.74, *P* < .0001). A high PG performer was 2.5 times more likely to begin with an open-ended question like “How can I help?” than a low PG performer (RR = 2.50, 95% CI: 1.69-3.70, *P* < .0001). Being a high PG performer was associated with the positive behavior of doing at least 1 nonmedical gesture 4.13 times more than a low performer (RR = 4.13, 95% CI: 2.57-6.61, *P* < .0001). A high PG performer was 6.88 times more likely to overestimate time than a low PG performer was (RR = 6.8, 95% CI: 3.70-12.78, *P* < .0001).

Table 1 demonstrates comparison of 8 PatientSET-positive behaviors between low PG performers and high PG performers. High PG performers exhibited a significantly higher frequency of 6 of the 8 behaviors, including (1) smile and make eye contact (RR = 1.55), (2) shake hands (RR = 1.56), (3) acknowledge the wait and apologize for it (RR = 16.76), (4) begin with open-ended question like “How can I help you?” (RR = 2.5), (5) Do at least 1 nonmedical gesture (RR = 4.13), and (6) Overestimate time (RR = 6.88).

**Figure 1.**
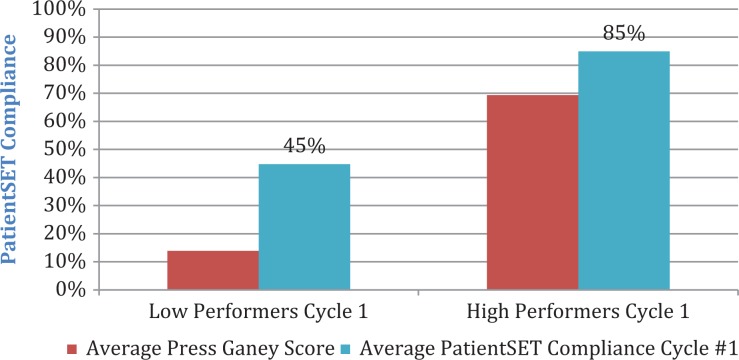
Overall PatientSET behavioral comparison of low versus high Press Ganey (PG) performers.

The mean overall observation score of 45% (standard deviation [SD] = 16.7%) for clinicians in the low PG performing group was significantly lower (*P* < .0001) than the mean overall observation score of 85% (SD = 15.6%) for clinicians in the high PG performing group, as per Figure 1.

## Discussion

Unlike prior communication studies that focused on physicians in training, this study measured the communication behaviors of practicing clinicians in a real-time ED setting. Our observational data showed a positive correlation between high performing ED clinicians in PG satisfaction scores and use of the following 6 PatientSET communication behaviors:

1. Smile and make eye contact.
2. Shake hands.
3. Acknowledge the wait and apologize for it.
4. Begin with open-ended question like “How can I help you?”
5. Do at least 1 nonmedical gesture.
6. Overestimate time.

The ED clinicians seeking to maximize their PG satisfaction scores might consider these behaviors. In our study, the concept of appreciation of the patient’s time showed the most significant behavioral differences between the high and low PG performers. This included the behaviors “acknowledge the wait” and “apologize for it” (RR = 16.76) and “overestimate time” (RR = 6.88). An overestimate of time was observed to be present when the clinician verbalized a time estimate to the patient.

Nonmedical gestures included but not limited to giving the patient a pillow or blanket, adjusting their bed for comfort, getting them food or drink, or asking if they could do anything to make them more comfortable. Among our ED clinicians, the high PG performers consistently performed these positive PatientSET behaviors in comparison to the low PG performers. The 2 behaviors that did not vary among the high versus low PG performers were “pause before entering” and “introduce self and others.”

A limitation in our study may have been that ED clinicians knew they were being observed for their communication skills during their initial interaction with ED patients. This awareness may have led to a Hawthorne effect, with the frequency of positive behaviors occurring more often than during the nonobserved patient encounters. Nevertheless, the low PG performers demonstrated the 8 positive bedside behaviors only 44.8% of the time while being observed for their “bedside manner,” a surprisingly low baseline frequency despite a potential Hawthorne bias.

Additionally, the use of PG scores may imply that it was the “gold standard” for evaluation of PS scores; however, such a contextual gold standard does not exist. Press Ganey is the selected survey instrument previously chosen by our hospital. It has been shown that subjective comments in PG surveys (not included in objective PG scores) can be determined through text mining for a more complete analysis of the patient’s rating of their experience (8,9). Furthermore, as each ED practice setting and patient population differs, our arbitrary cutoff of 40th percentile for high versus low performers may not be generalizable, as this was a single-site study.

Limitations also include the lack of inter-rater reliability amongst the observers. Although each observer prepared similarly with online training, the quality of his or her measurements may have varied. In addition, the observed presence or absence of the communication behaviors was binary and did not measure the quality of such behaviors. Lastly, the observation time occurred during the initial patient encounter, so observers could not verify the accuracy of time estimates given to patients.

Lastly, one of our authors created the observation tool chosen for this study. This biased the selection of this tool versus other communication tools.

The strength of our study was determining specific ED clinician communication behaviors that correlated with higher PS scores. There is a lack of literature about clinician behaviors that improve PS scores in the clinical setting. Unlike prior studies that focused on communication training prior to an attending status, our study reviewed behaviors of practicing ED clinicians in real time.

This study is a novel addition to the health-care communication literature. As we consider training across all clinician groups beginning with medical students, this may enhance our knowledge of what specific communication behaviors may improve PS scores. Considering the current state of health-care institutions competing for patients as well as centers for medicare and medicaid services reimbursement from PS, this information may help focus clinician communication training efforts.

## Conclusion

To our knowledge, this is the first time that specific ED clinician communication behaviors have been measured for correlation with PS scores. The 6 communication behaviors (1) smile and make eye contact, (2) shake hands, (3) acknowledge the wait and apologize for it, (4) begin with open-ended question like “How can I help you?”, (5) do at least 1 nonmedical gesture, and (6) overestimate time were found to be more prevalent in our higher performing PG group. These behaviors may be important for ED clinicians seeking to achieve higher PS scores. Further study is needed to prospectively follow clinician PG scores after such training to better assess the effect of training intervention and PG scores.

We conclude that specific positive communication behaviors may be associated with ED clinicians with higher PG scores. In addition, a communication training program like the PatientSET program may assist clinicians seeking to utilize these specific communication behaviors. The ED clinicians may incorporate these specific 6 communication behaviors to improve their PG scores.
